# Prevalence and Awareness of Hypertension Among Older Adults

**DOI:** 10.7759/cureus.85622

**Published:** 2025-06-09

**Authors:** Ambreen Ambreen, Muhammad Farooq, Zainab Abdul Islam, Hira Akhtar, Syeda Khulood Fatima, Hamid Ullah

**Affiliations:** 1 Internal Medicine, Shifa International Hospital, Faisalabad, PAK; 2 Medicine, Tertiary Care Hospital Nishtar-II/Nishtar Medical University (NMU), Multan, PAK; 3 Medicine, Bahria University Medical and Dental College, Karachi, PAK; 4 Dermatology, Bahawal Victoria Hospital, Bahawalpur, PAK; 5 Quality Management, Behzad Medical Est., Sitra, BHR; 6 Medicine, Bacha Khan Medical College, Mardan, PAK

**Keywords:** awareness, hypertension, older adults, pakistan, prevalence

## Abstract

Background

One of the main preventable risk factors for cardiovascular disease is hypertension, which is becoming more common in older persons.

Objective

The objective of the study is to determine the prevalence of hypertension and assess the level of awareness regarding the condition among older adults aged 60 years and above.

Methodology

A descriptive cross-sectional study was conducted at Bacha Khan Medical College, Mardan, from January to December 2023. Using a convenience sample, 308 people who were at least 60 years old were recruited. Structured in-person interviews and blood pressure readings using a calibrated digital sphygmomanometer were used to gather data. Systolic blood pressure ≥ 140 mmHg, diastolic blood pressure ≥ 90 mmHg, or the use of antihypertensive medication were considered indicators of hypertension. Prevalence and awareness were calculated using descriptive statistics, and correlations between awareness and demographic characteristics were investigated using chi-squared tests.

Results

Hypertension was present in 61.36% of the population (n = 189). Of them, 30.69% (n = 58) were unaware of their condition, while 69.31% (n = 131) knew. The most prevalent kind of hypertension, affecting 46.75% (n = 144) of the sample as a whole, was stage 2; 75.13% (n = 142) of the hypertensive patients were taking antihypertensive medication. Education level (p < 0.001), age group (p = 0.024), and residency (p = 0.006) were all substantially correlated with awareness, but not gender (p = 0.411).

Conclusion

Hypertension is highly prevalent among older adults, with substantial gaps in awareness, especially among the less educated and rural populations.

## Introduction

One of the most widespread and enduring worldwide public health issues is hypertension, sometimes known as high blood pressure (BP) [1]. It is a major modifiable risk factor for stroke, heart disease, kidney failure, and early death [2]. The prevalence of hypertension rises dramatically with population age, especially in older persons who are more susceptible to its consequences because of comorbidities and age-related vascular alterations [3]. Many people are unaware that they have hypertension until major health issues arise, and the problem often advances quietly [4].

A significant percentage of elderly persons in many nations, particularly those with developed healthcare systems, suffer from undiagnosed or inadequately treated hypertension [5]. This highlights a wider problem of health literacy and awareness in addition to a gap in clinical identification and treatment [6]. The incidence of hypertension and the level of knowledge among the elderly may be influenced by a number of factors, including socioeconomic position, education level, access to healthcare facilities, and cultural beliefs [7].

The increased burden of undiagnosed hypertension in emerging nations is exacerbated by inadequate public health education initiatives, a lack of regular screening, and a lack of health infrastructure [8]. In Pakistan, hypertension affects more than one-third of the adult population, with prevalence increasing to over 55% among those aged 60 and above. Studies also show that awareness and treatment rates are disproportionately low among elderly individuals, particularly women and those from rural areas [9].

To guide preventative measures and enhance health outcomes, it is essential to comprehend the present state of hypertension prevalence and knowledge in older populations [10]. In nations experiencing demographic changes, where the percentage of elderly persons is continuously increasing, this is especially crucial [11]. Frequent tracking of these markers lowers the chance of difficulties from hypertension later in life by assisting legislators and healthcare professionals in filling in screening, education, and treatment gaps [12].

For the purpose of directing future public health initiatives and resource allocation, precise, population-specific statistics about the prevalence and knowledge of hypertension in older people are crucial. By investigating the present trends in hypertension and awareness among those 60 years of age and older, this research aims to support this endeavor. The objective of this study was to determine the prevalence of hypertension and assess the level of awareness regarding the condition among older adults.

## Materials and methods

Study design and setting

This was a descriptive cross-sectional study conducted at Bacha Khan Medical College, Mardan, over a period of one year from January 2023 to December 2023.

Inclusion and exclusion criteria

Participants aged 60 years and older were eligible. Informed consent was obtained from all participants, who were recruited either from hospital settings or through community outreach activities. The exclusion criteria included critically ill patients, individuals with cognitive impairment that hindered effective communication, and those who declined to participate.

Sample size

Due to logistical constraints such as limited time, staff, and financial resources, a convenience sampling strategy was used. A total of 308 older adults (aged ≥60 years) were enrolled. This sample size was deemed sufficient for estimating hypertension prevalence and awareness levels in the target population.

To validate sample adequacy, a post hoc power analysis was conducted using an observed hypertension prevalence of 69.3% based on a prior regional study among older adults in Pakistan [13]. Using a two-sided test at a 0.05 significance level, the analysis yielded a statistical power of 99.8%, confirming that the sample size was sufficient to detect a meaningful difference from a baseline prevalence of 50%, a conventional threshold in public health research.

Data collection

Data were collected through structured, face-to-face interviews using a standardized questionnaire (see the appendix). The tool comprised sections on sociodemographic variables, hypertension history, medication usage, knowledge of BP status, and barriers to treatment adherence. Hypertension classification followed the 2017 American College of Cardiology (ACC)/American Heart Association (AHA) guidelines [14], with stage 1 hypertension defined as systolic BP (SBP) 130-139 mmHg or diastolic BP (DBP) 80-89 mmHg and stage 2 hypertension defined as SBP ≥ 140 mmHg or DBP ≥ 90 mmHg.

BP was measured using a validated and calibrated digital sphygmomanometer by trained personnel. Each participant was seated with their back supported, feet flat on the floor, and legs uncrossed. After resting for at least five minutes, two readings were taken from the same arm, spaced five minutes apart, and the mean was used for analysis. Although BP was recorded during a single visit, this procedure is consistent with WHO STEPS [15] and AHA/European Society of Hypertension (ESH) [14] recommendations for epidemiological settings.

Questionnaire development and validation

The questionnaire was developed through a systematic, evidence-based approach. A literature review [16,17] informed the initial item pool, which was reviewed by a panel of experts including clinicians and epidemiologists. Revisions were made based on expert feedback to improve content clarity and relevance.

A pre-test was conducted with 30 older adults from the target population to assess comprehension, layout, and cultural appropriateness. Minor adjustments followed. Face validity was assessed through participant feedback. Reliability testing using Cronbach’s alpha yielded 0.85, indicating strong internal consistency. Construct validity was established via exploratory factor analysis, which confirmed the theoretical item structure. The final tool was translated into the local language and back-translated to ensure linguistic and contextual accuracy.

Statistical analysis

Data were analyzed using SPSS version 25 (IBM Corp., Armonk, NY, US). Descriptive statistics (means, SDs, frequencies, and percentages) were used to summarize the data. The Shapiro-Wilk test was applied to assess the normality of continuous variables such as age and BP. Non-parametric tests (e.g., Mann-Whitney U and Kruskal-Wallis) were used where appropriate [18]. We analyzed awareness and treatment rates stratified by hypertension stage. We also identified key barriers to medication adherence, including cost, side effects, lack of awareness, and irregular follow-up.

To assess independent predictors of hypertension awareness, a binary logistic regression was conducted. Variables with p < 0.2 in univariate analyses were included in the model. Model fit was verified using the Hosmer-Lemeshow test, and results are presented as adjusted odds ratios (AORs) with 95% confidence intervals (CIs) [19]. Chi-squared tests were used to assess associations between categorical variables. A p-value < 0.05 was considered statistically significant.

Ethical approval

The study protocol was reviewed and approved by the Institutional Ethical Review Committee of Bacha Khan Medical College, Mardan. Written informed consent was obtained from all participants. Data confidentiality and ethical standards were strictly maintained throughout the study.

## Results

The sociodemographic details of the 308 research participants are shown in Table 1. Most were between the ages of 60 and 69 (56.49%), followed by those between the ages of 70 and 79 (31.17%) and those over 80 (12.34%). The sample was composed of 47.40% women and 52.60% men. In terms of education, 24.68% had completed elementary school, 21.10% had completed secondary school, 17.53% had completed further education, and 36.69% were illiterate. The majority of participants (58.77%) lived in cities, while 41.23% did so in rural regions.

**Table 1:** Sociodemographic characteristics of study participants (n = 308)

Variable	Category	Number of patients (n, %)
Age group (years)	60–69	174 (56.49)
	70–79	96 (31.17)
	≥80	38 (12.34)
Gender	Male	162 (52.60)
	Female	146 (47.40)
Education level	Illiterate	113 (36.69)
	Primary	76 (24.68)
	Secondary	65 (21.10)
	Higher	54 (17.53)
Residence	Urban	181 (58.77)
	Rural	127 (41.23)

Table 2 categorizes participants based on the average of two BP measurements and further stratifies them by awareness and treatment status. Of the 308 participants, 54 (17.53%) had normal BP, 38 (12.34%) had elevated BP, 72 (23.38%) had stage 1 hypertension, and 144 (46.75%) had stage 2 hypertension. Awareness and treatment rates increased with hypertension severity: only 11.11% of those with normal BP and 23.68% with elevated BP were aware of their condition, compared to 38.89% in stage 1 and 61.11% in stage 2. Similarly, antihypertensive treatment was reported by 7.41% of individuals with normal BP and 13.16% of those with elevated BP, while 29.17% of stage 1 and 54.17% of stage 2 hypertensive participants were on medication. These findings highlight a clear gradient in disease recognition and treatment linked to BP severity.

**Table 2:** Stratify awareness and treatment by BP stage (n = 308) BP: blood pressure

BP category (mmHg)	Number of patients (n, %)	Aware of hypertension (n, %)	On antihypertensive treatment (n, %)
Normal (<120/80)	54 (17.53%)	6 (11.11%)	4 (7.41%)
Elevated (120–129/<80)	38 (12.34%)	9 (23.68%)	5 (13.16%)
Stage 1 hypertension (130–139/80–89)	72 (23.38%)	28 (38.89%)	21 (29.17%)
Stage 2 hypertension (≥140/≥90)	144 (46.75%)	88 (61.11%)	78 (54.17%)

**Figure 1:**
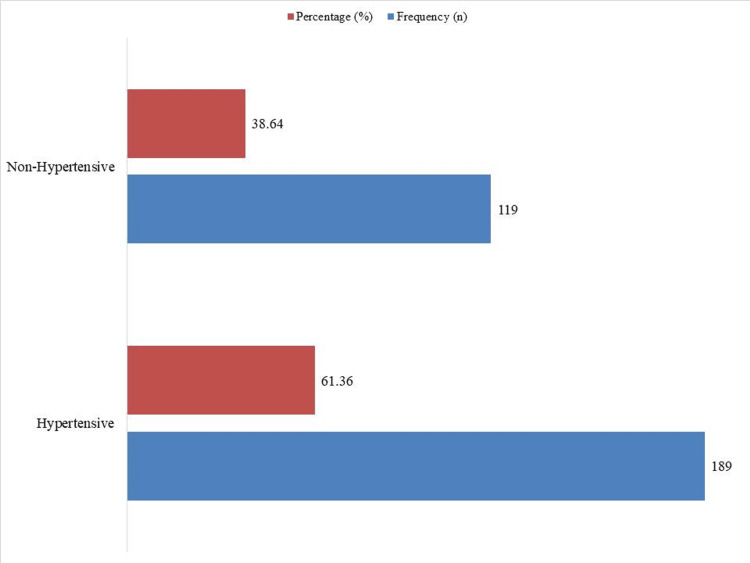
Prevalence of hypertension based on BP or medication use (n = 308) BP: blood pressure

The BP classifications in Table 2 are based solely on measured BP values obtained during the study, without considering participants' current use of antihypertensive medications. However, for the purpose of estimating the overall prevalence of hypertension in the population, a more inclusive definition was used in Figure 1. This definition includes individuals with elevated BP (≥140/90 mmHg) and those currently using antihypertensive medications, even if their measured BP was within normal range due to treatment. This approach provides a more clinically accurate estimate of true hypertension prevalence.

Figure 1 classifies participants as hypertensive or non-hypertensive based on both their BP readings and their use of antihypertensive medications. Using this combined criterion, 189 participants (61.36%) were classified as hypertensive-this group includes individuals with elevated BP and those who reported current use of antihypertensive medication, regardless of their measured BP at the time of the study. The remaining 119 participants (38.64%) had neither elevated BP nor reported use of antihypertensive drugs and were, thus, classified as non-hypertensive.

**Figure 2:**
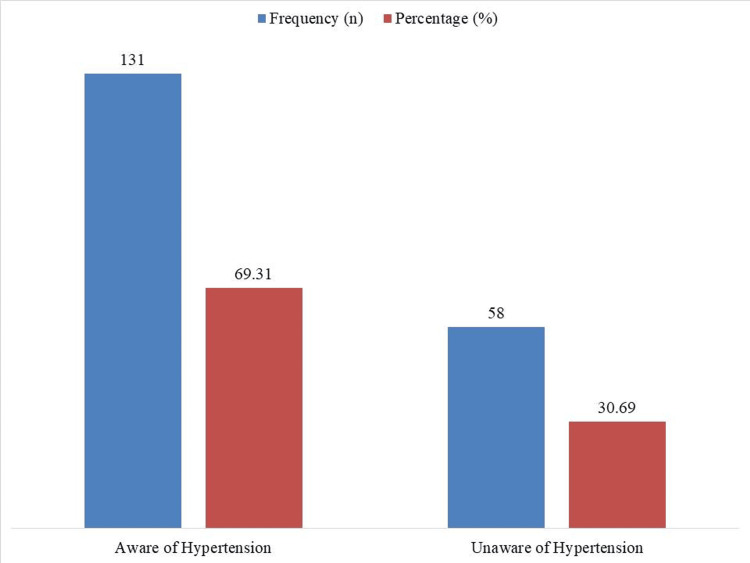
Awareness of hypertension among hypertensive participants (n = 189)

Figure 2 shows that there was a significant disparity in hypertension knowledge among the 189 hypertensive individuals, with 69.31% (n = 131) knowing of their disease and 30.69% (n = 58) not.

**Figure 3:**
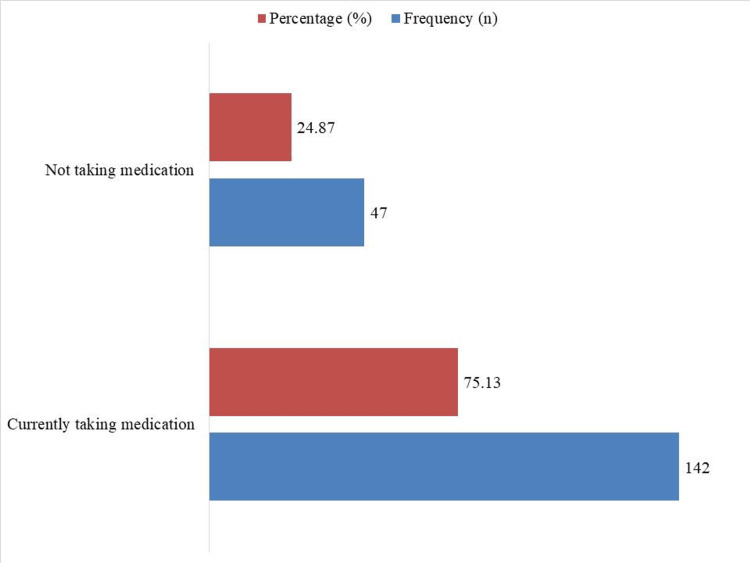
Antihypertensive medication use among hypertensive participants (n = 189)

According to Figure 3, 75.13% (n = 142) of participants with hypertension were presently taking antihypertensive medication, while 24.87% (n = 47) were not. This suggests that those who are aware of their disease are taking their medicine as prescribed.

Table 3 shows the association between hypertension awareness and demographic variables in 189 participants. Age group analysis revealed a significant difference in awareness among those aged 60-69 years (p = 0.024), while no significant differences were found in the older age groups. Gender had no significant impact on awareness (p = 0.411). Urban residents were significantly more aware of their hypertension compared to rural residents (p = 0.006). Education level had a strong association, with illiterate individuals showing a significant lack of awareness (p < 0.001), while those with primary, secondary, and higher education had fewer unaware individuals.

**Table 3:** Association between awareness of hypertension and demographic variables (n = 189)

Demographic variable		Aware (n)	Unaware (n)	Chi-squared value	p-value
Age group (years)	60–69	83	22	5.12	0.024
	70–79	39	17		
	≥80	9	19		
						Gender	Male	74	13	0.68	0.411
Female	57	45									
Residence	Urban	88	25	7.56	0.006
	Rural	43	33		
Education level	Illiterate	39	30	42.12	<0.001
	Primary	22	25		
	Secondary	35	5		
	Higher	29	4		

**Figure 4:**
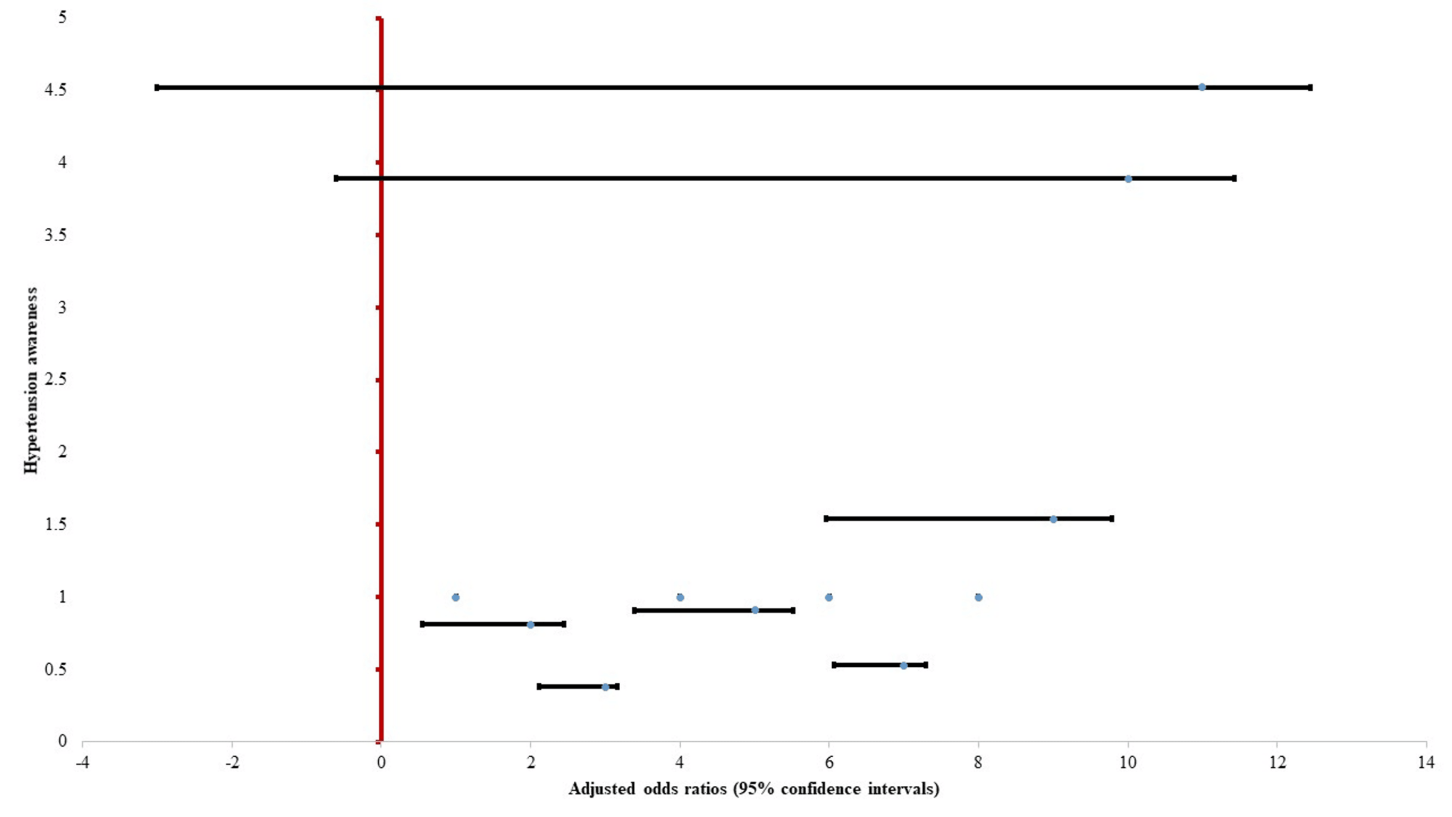
Multivariate logistic regression analysis for hypertension awareness Forest plot showing adjusted odds ratios (AORs) and 95% confidence intervals from multivariate logistic regression of predictors of hypertension awareness among hypertensive participants (n = 189). The vertical dashed line at AOR = 1 indicates no association. Variables with confidence intervals not crossing 1 are considered statistically significant.

Multivariate logistic regression identified several independent predictors of hypertension awareness (Figure 4). Compared to participants aged 60-69 years, those aged ≥80 had significantly lower odds of being aware of their condition (AOR = 0.38, 95% CI: 0.16-0.89, p = 0.026). Higher education was strongly associated with awareness; individuals with secondary (AOR = 3.89, p = 0.008) and higher (AOR = 4.52, p = 0.009) education had significantly increased odds of awareness compared to illiterate participants. Urban residence was also a significant predictor (AOR = 1.89, p = 0.028). Gender was not significantly associated with awareness.

## Discussion

According to the study's results, elderly individuals in this region bear an alarmingly high burden of hypertension. Based on direct BP measurements and/or current use of antihypertensive medication, 61.36% (n = 189) of the 308 participants were classified as hypertensive. This finding is consistent with previous research; for instance, one study reported a prevalence of 58.1% among adults aged ≥60 years in a regional sample [20], while a Chinese study found a slightly lower prevalence of 50.9% in the same age group [21]. Differences in prevalence rates may reflect variations in access to care, lifestyle patterns, and population health profiles.

Further analysis revealed a concerning distribution of BP severity: only 17.53% of participants had normal BP, while 12.34% had elevated BP, 23.38% had stage 1 hypertension, and 46.75% had stage 2 hypertension. The predominance of stage 2 hypertension suggests that many older adults remain undiagnosed or undertreated for prolonged periods. These findings mirror trends in other South Asian populations, where stage 2 hypertension has been shown to be the most common category among the elderly [22].

Encouragingly, awareness among hypertensive individuals was relatively high, with 69.31% (n = 131) acknowledging their condition. This is notably better than prior studies, where awareness rates among older hypertensive adults were reported as low as 45% [12,23]. Moreover, 75.13% (n = 142) of those diagnosed reported taking antihypertensive medication-significantly higher than treatment rates seen in prior studies from Turkey (31%-47%) [24]. This could reflect the impact of urban health programs, greater healthcare access, or family support systems in managing chronic diseases among older populations.

Importantly, awareness and treatment were strongly influenced by education level. Participants with higher education had an awareness rate of 88.24%, while those who were illiterate had only 44.26% awareness. This association aligns with existing literature highlighting the link between literacy and chronic disease management [25]. Similar disparities were observed across places of residence: urban participants were significantly more aware (67.18%) compared to rural participants (56.58%), which reflects well-documented urban-rural health inequities [26,27]. Additionally, younger seniors (60-69 years) demonstrated greater awareness than those aged ≥80, suggesting that age-targeted strategies may be required for the oldest segments of the population.

Policy implications and intervention strategies

This study highlights the significant prevalence of undiagnosed and untreated hypertension in older persons, necessitating immediate public health intervention. An integrated intervention strategy is essential to tackle inequities in awareness and treatment, especially among rural, uneducated, and elderly populations.

A suggested option is the implementation of mobile health clinics in underserved rural regions. These mobile machines can offer routine BP assessments, health counseling, and preliminary treatment services. Mobile clinics can alleviate geographic barriers that lead to diminished knowledge and untreated hypertension in rural populations by delivering healthcare directly to the community, particularly in underserved or remote areas.

An alternative effective strategy entails the utilization of community health workers (CHWs), who can function as primary agents of transformation. Trained CHWs can perform home-based BP monitoring, educate families on disease management, and offer follow-up assistance to enhance treatment adherence. This technique is especially advantageous for senior folks who may have mobility difficulties or necessitate culturally appropriate communication.

Moreover, health education programs must be customized to address the requirements of illiterate or semi-literate populations. Conventional print materials frequently prove ineffectual in these communities. Visual tools, audio communications through radio, and community theater or role-play techniques in local languages can be utilized instead. Culturally tailored educational initiatives can markedly enhance awareness and promote proactive health behaviors.

Particular emphasis should be placed on the oldest demographic (aged ≥80), which exhibited the lowest awareness rates in this study. This subgroup may benefit from streamlined instructional resources, caregiver participation in consultations, and regular home visits by healthcare professionals. These therapies mitigate cognitive or sensory deterioration and facilitate continuity of care.

Ultimately, policy initiatives should focus on enhancing the accessibility of antihypertensive drugs via subsidy programs or their incorporation into national essential drug lists. Cost continues to be a significant obstacle to sustained adherence, particularly among older persons with restricted resources. Guaranteeing accessible therapy is essential for enhancing results in this at-risk population. These interventions should be incorporated into Pakistan's comprehensive non-communicable disease control strategy, prioritizing early diagnosis, healthcare accessibility, and community-oriented prevention.

Strengths and limitations

This study provides significant insights into the prevalence and awareness of hypertension among older persons, a commonly overlooked and vulnerable demographic in public health research. A primary strength is the application of recognized clinical criteria for diagnosing hypertension, which includes direct BP measurements obtained through standardized methods. To improve reliability, two measurements were taken at five-minute intervals while individuals were seated at rest, and the average was utilized for analysis. Interviewer-administered questionnaires conducted in person enhanced data accuracy, especially beneficial for senior participants who may encounter difficulties with self-administered forms due to sensory, cognitive, or literacy constraints. The inclusion of urban and rural participants enhances the dataset's breadth, facilitating a more contextually diverse comprehension of the older population in the region. The study employed instrument preparation, encompassing pre-testing, expert validation, and psychometric assessment, and utilized multivariate logistic regression to account for factors affecting hypertension awareness, thereby augmenting the analytical rigor of the results.

However, we must acknowledge certain limitations. Convenience sampling restricts the generalizability of the findings to the wider older demographic. Despite efforts to standardize measures, dependence on single-visit BP tests raises the risk of misclassification due to the white-coat effect or transient variations. The cross-sectional design also prevents any inference of causality. Self-reported information concerning medicine utilization and knowledge is susceptible to recall bias. Ultimately, the study was performed in a singular institution, perhaps constraining the depiction of many geographic and cultural contexts throughout Pakistan.

## Conclusions

According to this research, hypertension is quite common among older folks, and a significant percentage of those who have it are not aware that they have it. Education level and urban residency were strongly correlated with awareness and treatment rates, underscoring the importance of socioeconomic variables in the identification and control of illness. These results call attention to the urgent need for community-based screening, education, and focused interventions to enhance the identification and management of hypertension in older adults.

## Appendices

Questionnaire

Section 1: Sociodemographic Information

Age Group:

60-69 years

70-79 years

80+ years

Gender:

Male

Female

Education Level:

Illiterate

Primary School

Secondary School

Higher Education

Residence:

Urban

Rural

Section 2: Medical History

Have you ever been diagnosed with hypertension by a healthcare provider?

Yes

No

Are you currently taking any antihypertensive medication?

Yes

No

Have you been informed by a healthcare provider that you have high blood pressure (hypertension)?

Yes

No

Have you ever had a blood pressure measurement taken?

Yes

No

If yes, when was your last blood pressure measurement?

Within the last 6 months

Within the last year

More than a year ago

Never had it measured

Section 3: Awareness of Hypertension

Do you know what hypertension (high blood pressure) is?

Yes

No

If yes, can you briefly explain what hypertension is?

________________________________________________________

Are you aware of the normal range for blood pressure?

Yes

No

Do you know the risks associated with untreated high blood pressure (e.g., heart disease and stroke)?

Yes

No

Do you follow any lifestyle changes or take any actions to manage your blood pressure (e.g., diet, exercise, and medication)?

Yes

No

Have you ever attended a health education session or screening program for hypertension?

Yes

No

Do you think that hypertension is common among older adults?

Yes

No

Section 4: Blood Pressure Readings (Measured During Study)

Current blood pressure reading (to be recorded by the interviewer using a calibrated sphygmomanometer):

Systolic (mmHg): ________

Diastolic (mmHg): ________

Based on the blood pressure reading, do you fall into any of the following categories? (For interviewer only)

Normal (<120/80 mmHg)

Elevated (120-129/<80 mmHg)

Stage 1 Hypertension (130-139/80-89 mmHg)

Stage 2 Hypertension (≥140/≥90 mmHg)

Section 5: Health-Seeking Behavior and Treatment

Do you visit a healthcare provider regularly for check-ups?

Yes

No

If yes, how often do you visit the healthcare provider for general check-ups?

Once a year

Twice a year

More than twice a year

Less than once a year

If you are currently taking antihypertensive medication, do you follow the prescribed dosage regularly?

Yes

No

Not Applicable

Have you experienced any side effects from antihypertensive medication?

Yes

No

Not Applicable

If you have not been diagnosed with hypertension, do you plan to check your blood pressure in the future?

Yes

No

Unsure
